# Plasmids are vectors for redundant chromosomal genes in the *Bacillus cereus* group

**DOI:** 10.1186/s12864-014-1206-5

**Published:** 2015-01-22

**Authors:** Jinshui Zheng, Ziyu Guan, Shiyun Cao, Donghai Peng, Lifang Ruan, Daohong Jiang, Ming Sun

**Affiliations:** State Key Laboratory of Agricultural Microbiology, Huazhong Agricultural University, Wuhan, 430070 China

**Keywords:** Plasmid, Chromosome, Pan-genome, *Bacillus cereus* group

## Abstract

**Background:**

Prokaryotic plasmids have played significant roles in the evolution of bacterial genomes and have a great impact on the metabolic functions of the host cell. Many bacterial strains contain multiple plasmids, but the relationships between bacterial plasmids and chromosomes are unclear. We focused on plasmids from the *Bacillus cereus* group because most strains contain several plasmids.

**Results:**

We collected the genome sequences of 104 plasmids and 20 chromosomes from *B. cereus* group strains, and we studied the relationships between plasmids and chromosomes by focusing on the pan-genomes of these plasmids and chromosomes. In terms of basic features (base composition and codon usage), the genes on plasmids were more similar to the chromosomal variable genes (distributed genes and unique genes) than to the chromosomal core genes. Although all the functional categories of the chromosomal genes were exhibited by the plasmid genes, the proportions of each category differed between these two gene sets. The 598 gene families shared between chromosomes and plasmids displayed a uniform distribution between the two groups. A phylogenetic analysis of the shared genes, including the chromosomal core gene set, indicated that gene exchange events between plasmids and chromosomes occurred frequently during the evolutionary histories of the strains and species in this group. Moreover, the shared genes between plasmids and chromosomes usually had different promoter and terminator sequences, suggesting that they are regulated by different elements at the transcriptional level.

**Conclusions:**

We speculate that for the entire *B. cereus* group, adaptive genes are preserved on both plasmids and chromosomes; however, in a single cell, homologous genes on plasmids and the chromosome are controlled by different regulators to reduce the burden of maintaining redundant genes.

**Electronic supplementary material:**

The online version of this article (doi:10.1186/s12864-014-1206-5) contains supplementary material, which is available to authorized users.

## Background

Horizontal gene transfer (HGT) plays an important role in bacterial evolution by providing foreign genetic material for gene exchange between prokaryotes [1]. One of the most important contributors to HGT is plasmids, which can be transferred between cells as vectors for genes and can provide a basis for genomic rearrangements via homologous recombination [2]. In this process, events in which genes are gained and/or lost force bacterial genomes to evolve. Moreover, many adaptive genes contained by plasmids are transferred, and these genes play important roles in bacterial adaptation to changing environments [3,4].

Plasmids have been studied for different purposes by many researchers. These studies have mainly focused on the intrinsic characteristics and accessory functions of plasmids. Among the former topics, plasmid replication, maintenance and mobilization have been the major subjects [5,6]; among the latter, contributions to antibiotic resistance (AR) and virulence have been the primary concerns [7]. Recently, as increasing numbers of plasmid genomic sequences have become available, systematic analyses of the dynamics and relationships among plasmids and their contributions to bacterial genomic evolution have become feasible. Tamminen et al. used network methods to study all of the 2,343 plasmids with available genomic sequences and described these plasmids’ evolutionary dynamics and interrelationships [8]. By analyzing the plasmids of genus *Acinetobacter*, the same research group found that although most of these plasmids lack mobilization and transfer functions, they likely have a long history of rearrangements with other plasmids and with chromosomes [9]. Moreover, other research has revealed that plasmids have played more important roles than viruses in the evolution of bacterial genomes [10]. In addition to mediating HGT among different bacterial cells, plasmids contribute to bacterial evolution via their role in the formation and propagation of operons, a process in which plasmids have been likened to scribbling pads [11].

Because plasmids coexist with chromosomes in bacterial cells, the relationships between plasmids and chromosomes are critical for understanding the evolution and diversity of bacterial genomes. These relationships have been directly studied by focusing on gene exchange events between plasmids and chromosomes. Such events can be caused by transposons, phages, integrons and plasmids [12-15]. In addition, transposons and integrons can be found on both plasmids and chromosomes, and phages can be integrated into chromosomes and plasmids as prophages [16-18]. Even plasmids have been found to frequently integrate into chromosomes as integrative and conjugative elements [19]. However, no systematic analysis has closely examined the relationships between plasmids and chromosomes on a genome-wide scale. For example, in a particular species, how do plasmids affect chromosomal structures, what is the frequency of genetic exchange events between plasmids and chromosomes, and why are some genes harbored by both plasmids and chromosomes? In a previous study, we used the *Bacillus cereus* group as a model to explore the evolution and dynamics of plasmids [20]. In the present study, we use the *B. cereus* group as a model to study the relationships between plasmids and chromosomes by focusing on the genes that are shared between them.

Members of the *B. cereus* group are found in diverse environments, including soil, water, and animal hosts, and they include species of *B. anthracis*, *B. cereus*, *B. thuringiensis* and four more variable species, *B. cytotoxicus*, *B. mycoides*, *B. pseudomycoides*, and *B. weihenstephanensis* [21,22]. Plasmids are important for defining the first three species [21,23,24]. The plasmids in this group display strain-dependent distribution, with some strains containing no plasmids, whereas others have many (more than 10) [25-27]. Some of these plasmids have small genome sizes, only 2 kb [28], whereas others are very large, up to 600 kb. Even within the same cell, the genome sizes of different plasmids vary widely; for example, *B. thuringiensis* CT-43 has 10 plasmids with genome sizes ranging from 6 kb to 300 kb [26]. In our recent work, we found that megaplasmids larger than 100 kb may have originated from integration events of smaller plasmids [20]. Furthermore, as reported previously, the total amount of plasmid DNA in a single *B. thuringiensis* cell is greater than that of chromosomal DNA [29]. This finding raises a question: What is the nature of the relationship between plasmids and the chromosome?

We studied the relationships between chromosomes and plasmids by focusing on their shared genes. Clusters of orthologous groups (COGs) and base composition analyses indicated that plasmids may contain an additional copy of a variable chromosomal region. We also examined genetic exchanges between plasmids and chromosomes by focusing on the basic features of their shared genes.

## Results

### Plasmids of the *B. cereus* group share dynamic gene pools with chromosomes

We focused on pan-genomic plasmids and chromosomes to study the relationships between plasmids and chromosomes. The numbers of MCL (Markov Cluster) family members obtained using the OrthoMCL tool for the chromosomal core gene set (genes shared by all of the 20 chromosomes), chromosomal distributed gene set (genes shared by more than one chromosome but less than 20), chromosomal unique gene set (all the individual genes present on only one chromosome), plasmid distributed gene set (genes shared by more than one plasmid) and plasmid unique gene set (all the individual genes present on only one plasmid) were 2009, 3933, 6813, 1121 and 4934, respectively. There were no core genes shared by all the plasmids. Overall, there were 598 gene families shared by plasmids and chromosomes.

**Figure 1:**
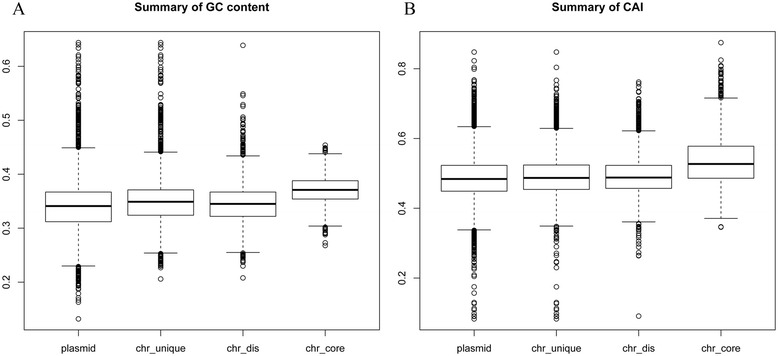
**Basic features of genes from plasmids and chromosomes. (A)** Summary of the GC content of different data sets. **(B)** Summary of the CAI of different data sets. Plasmid, genes on plasmids; chr_unique, unique genes on chromosomes; chr_dis, distributed genes on chromosomes; chr_core, core genes on chromosomes.

We compared the basic features of genes from plasmids and chromosomes by analyzing the base composition of the gene sets described above. The average GC content of the genes on plasmids (34.1%) was more similar to that the two types of variable genes (34.9% for chromosomal unique genes and 34.5% for chromosomal distributed genes) than to that of the chromosomal core genes (37.1%) (P = 0.48, 0.32 and 2.3 × 10^−6^, Mann–Whitney test) (Figure 1A). A codon usage analysis with CAI (codon adaptation index) indicated that the plasmid genes showed no difference from the chromosomal variable genes (the P values for the plasmid genes compared with the two types of variable genes were 0.05 and 0.55, respectively; Mann–Whitney test) but were significantly different from the chromosomal core genes (P < 2.2 × 10^−16^, Mann–Whitney test) (Figure 1B). This finding indicates that the genes on plasmids share similar features with the variable genes (distributed genes and unique genes) of chromosomes, and the plasmids and chromosomes share the same dynamic gene pool.

**Figure 2:**
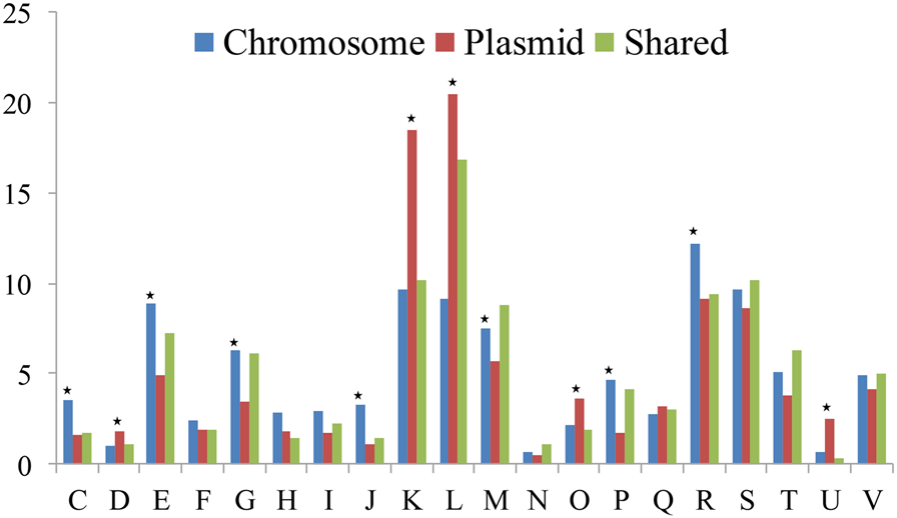
**Proportions of each COG category for all types of gene sets.** C, Energy production and conversion; D, Cell cycle control and mitosis; E, Amino acid metabolism and transport; F, Nucleotide metabolism and transport; G, Carbohydrate metabolism and transport; H, Coenzyme metabolism; I, Lipid metabolism; J, Translation; K, Transcription; L, Replication and repair; M, Cell wall/membrane/envelope biogenesis; N, Cell motility; O, Post-translational modification, protein turnover, and chaperone functions; P, Inorganic ion transport and metabolism; Q, Secondary structure; T, Signal transduction; R, General functional prediction only; S, Function unknown; U, Intracellular trafficking and secretion; V, Defense mechanisms.

We focused on functions determined by plasmids and found that all of the COG categories represented by chromosomes could be found on plasmids (Figure 2). By computing the proportion of gene families for each COG functional category, we found that approximately one-third of all the COG categories showed similar distributions on plasmids and chromosomes. The other two-thirds of the COG categories exhibited different distribution characteristics between plasmids and chromosomes. Gene families involved in replication, recombination, and repair represented the largest proportion of plasmid genes, but they occupied a significantly smaller proportion of the chromosomes (P < 2.2 × 10^−16^, one-sided binomial test). Moreover, gene families involved in transcription were also significantly more enriched on plasmids than on chromosomes (P < 2.2 × 10^−16^). On plasmids, these two types of gene families constituted almost half of the total gene families with known COG annotations. Other gene families, such as those involved in posttranslational modifications, protein turnover, chaperoning (P = 2.83 × 10^−5^) and intracellular trafficking, secretion, and vesicular transport (P = 1.12 × 10^−13^), were also enriched on plasmids. Conversely, the proportion of gene families involved in basal metabolism, such as those involved in amino acid transport and metabolism (P = 6.08 × 10^−11^), carbohydrate transport and metabolism, lipid metabolism (P = 3.34 × 10^−8^), inorganic ion transport and metabolism (P = 1.24 × 10^−11^) and energy production and conversion (P = 1.77 × 10^−6^) was significantly lower on plasmids than on chromosomes. In addition, gene families involved in translation were significantly more frequently found on chromosomes than on plasmids (P = 1.37 × 10^−9^).

### Genetic exchange events between plasmids and chromosomes have occurred frequently during the evolutionary history of the *B. cereus* group

**Figure 3:**
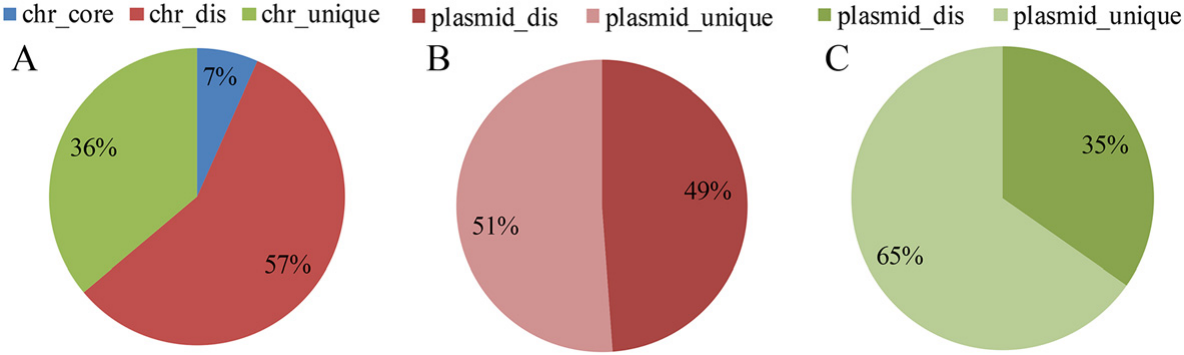
**The 598 gene families shared by plasmids and chromosomes were distributed over all the categories of the chromosomal gene set.** Each of the three gene sets contained shared genes **(A)**. Shared genes from the distributed set were shared by one and more than one plasmid equally **(B)**. Two-thirds of the shared genes from the chromosomal unique set were shared by one plasmid **(C)**.

Shared genes (homologous DNA fragments) between plasmids and chromosomes are the result of genetic exchange events. The 598 gene families shared by plasmids and chromosomes were found to be distributed across all the categories of the chromosomal gene set. For chromosomes, the largest number of shared genes was found in the distributed gene set, which included 342 gene families. The second largest number was in the unique gene set, which had 216 families, and the remainder was in the core gene set (Figure 3A). Equal numbers of shared genes from the distributed set were shared by one or multiple plasmids (Figure 3B), indicating that both single and multiple genetic exchange events among plasmids and chromosomes occurred during evolutionary history. Two-thirds of the shared genes from the chromosomal unique set were shared by one plasmid (Figure 3C); these may have resulted from recent genetic exchanges.

Genetic exchange events between chromosomes and plasmids were not restricted to certain regions; we found a uniform distribution of these shared genes throughout the chromosome. For example, the distribution of shared genes on the chromosome of *B. anthracis* Ames Ancestor showed no significant difference from a uniform random distribution (P = 0.34, Mann–Whitney test). The same result was observed for plasmids, which generally exhibited uniformly located shared genes (for pBMB171, P = 0.42, Mann–Whitney test).

The numbers of shared genes between a given plasmid and different chromosomes varied greatly. We found that among the 20 genomes studied, the plasmid/ chromosome pairs with the greatest number of shared genes were never in the same cell. The most extreme example was the plasmid pBWB401 from a *B. weihenstephanensis* strain. This plasmid shared fewer than 50 genes with most of the *B. cereus* group chromosomes, but it shared 93 genes with *B. cereus* B4264. In fact, this plasmid and chromosome pair shared a DNA fragment of 105 kb (base pairs 3,422,398–3,528,167 of the *B. cereus* B4264 chromosome), including 57 coding sequences, with an average nucleotide sequence identity greater than 95%. A recent genetic exchange may have occurred between pBWB401 and the *B. cereus* chromosome, after which the plasmid and the chromosome were separated.

Many genetic exchange events involved multiple genes (Additional file 1: Table S3). When the 57 uninterrupted genes shared by plasmid pBWB401 and chromosome *B. cereus* B4264 were excluded, 155 (29%) of the 541 genes shared by plasmids and chromosomes constituted 58 operons. The smallest operon consisted of 2 genes, and the largest contained 9 genes. The genes in the same operon exhibited functional relatedness.

**Figure 4:**
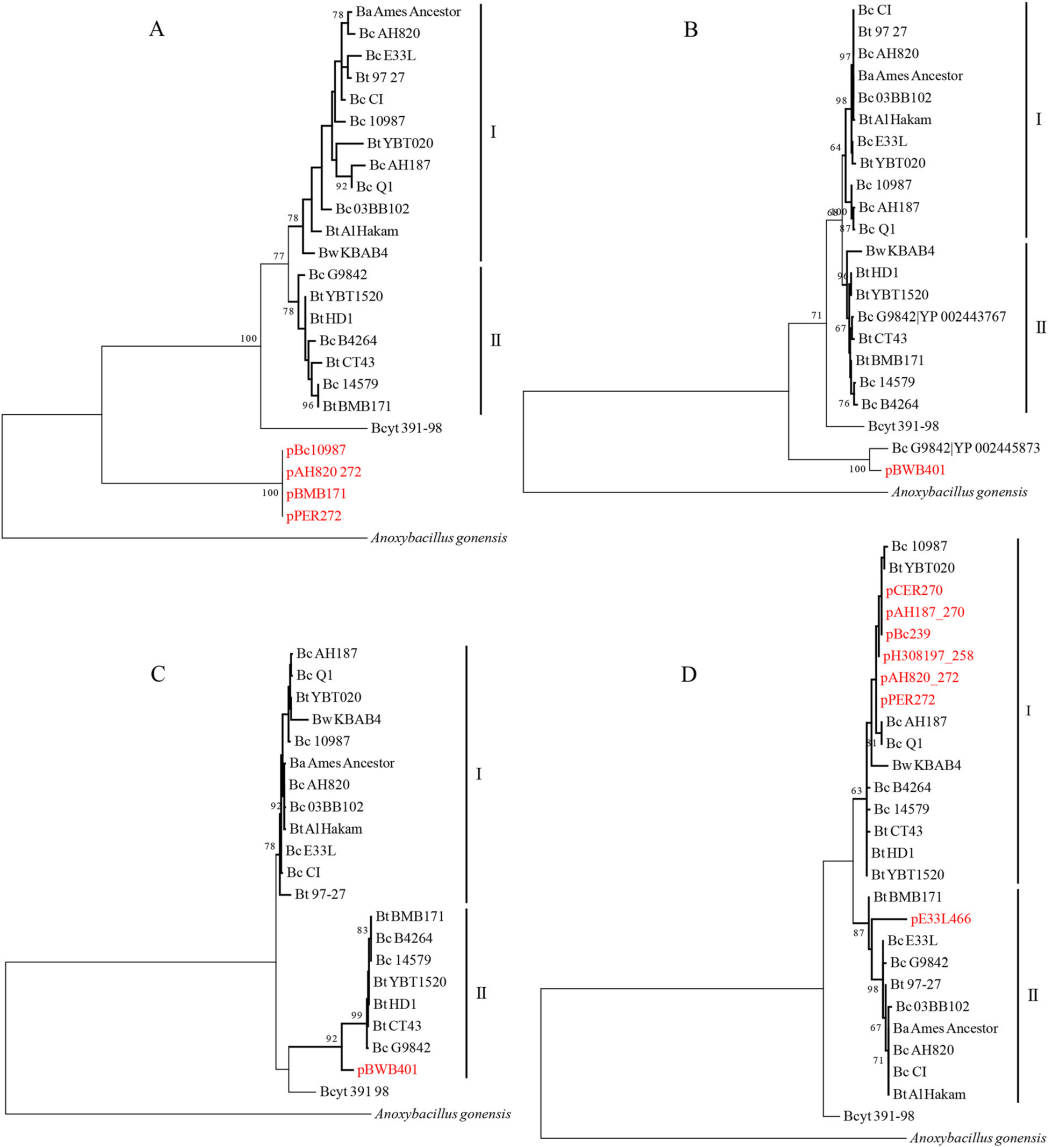
**Phylogenetic analysis based on the protein sequences of the shared genes between plasmids and the chromosomal core gene set. (A)** Shared genes on plasmids appeared as an outgroup from their homologous chromosomal core genes. **(B)** Shared genes on plasmids and one copy of the chromosome appeared as outgroups from their homologous chromosomal core genes. **(C)** Shared genes on plasmids were clustered with one of the lineages based on their homologous chromosomal core genes. **(D)** Shared genes on plasmids were distributed in both lineages based on their homologous chromosomal core genes. Homologous protein sequences from *Anoxybacillus gonensis* were used as an outgroup for the homologies from the entire *B. cereus* group. Lineages I and II were defined as in our previous work [30]. The number at each branch point represents the percentage of bootstrap support calculated from 100 replicates, and only those values higher than 60 are shown.

Genetic exchange events occurred frequently during the evolutionary histories of the members of the *B. cereus* group. Of the 40 shared genes of the chromosomal core gene set, 19 were exchanged between chromosomes and plasmids during the formation of the species; these 19 genes appeared as outgroups to the chromosomal homologous genes on the phylogenetic trees. For 13 of these 19 genes, the exchange events occurred only on plasmids after the different species’ lineages had formed; there was no evidence of recent homologous recombination with chromosomal genes (see example in Figure 4A). The other 6 genes were frequently exchanged between plasmids and chromosomes, and some duplication of genes on chromosomes was caused by these events (see example in Figure 4B). Among the 11 shared genes that were exchanged by plasmids after the formation of *B. cereus* group lineages, some were from lineage I or II (see example in Figure 4C) and others on different plasmids had different sources (see example in Figure 4D).

When the COG functions of the shared genes were analyzed, we found that the genes that were most frequently shared among different plasmids were those that function as transporters. As shown in Table 1, among the 55 shared genes with known COG annotations in the chromosomal core and extended core gene sets (genes shared by more than 19 chromosomes), 16 were annotated as transporters. Additionally, among the 18 genes with known COG annotations that were shared by more than 10 plasmids, 7 were transporter-associated genes. The gene that was shared by the most chromosomes and plasmids was a transporter-associated gene encoding the substrate-binding component of an ABC-type oligopeptide import system containing type 2 periplasmic binding folds. Other shared genes frequently contained in the chromosomal core and extended core sets were annotated as enzymes that participate in carbon and nitrogen metabolism. Among the 216 shared genes from the chromosomal unique set, the predominant functions were related to recombination; 17 and 7 genes were predicted to encode transposases and resolvases, respectively.

**Table 1:** **COG annotations of the shared genes among the chromosomal core and extended core sets**

**COG ID**	**Functional annotation**	**Chromosome number**	**Plasmid number**
32477	Predicted membrane protein [Function unknown]	20	7
131886	Stage V sporulation protein AE	20	6
131885	Stage V sporulation protein AC	20	6
183504	Stage V sporulation protein AD	20	6
178955	ATP-dependent Clp protease proteolytic subunit	20	5
105987	Hypothetical protein	20	5
193180	MacB-like periplasmic core domain.	20	4
73014	This family is composed of MJ0796 ATP-binding cassette, macrolide-specific ABC-type efflux carrier (MacAB), and proteins involved in cell division (FtsE) and release of lipoproteins from the cytoplasmic membrane (LolCDE)	20	4
162057	Arsenic-resistance protein	20	4
31088	Response regulators consisting of a CheY-like receiver domain and a winged-helix DNA-binding domain	20	4
162505	RND family efflux transporter, MFP subunit	20	4
115457	Sugar transport protein	20	4
184117	Arsenate reductase	20	4
181585	Glucose-1-dehydrogenase	20	4
32652	Zn-ribbon-containing protein involved in phosphonate metabolism	20	3
163006	Polysaccharide deacetylase family sporulation protein PdaB	20	3
31331	ABC-type antimicrobial peptide transport system, ATPase component	20	3
31475	Uncharacterized conserved protein	20	3
197627	Methyl-accepting chemotaxis-like domains (chemotaxis sensory transducer)	20	3
190390	FtsX-like permease family	20	3
179411	Adenine phosphoribosyltransferase	20	2
31326	ABC-type multidrug transport system, ATPase component	20	2
32452	Sugar phosphate permease	20	1
151609	Protein of unknown function	20	1
188197	Penicillin-binding protein, 1A family	20	1
34374	Predicted membrane protein	20	1
31911	Predicted transcriptional regulators	20	1
189896	Formate/nitrite transporter	20	1
34876	Uncharacterized protein involved in cytokinesis, contains TGc (transglutaminase/protease-like) domain	20	1
30931	Uncharacterized membrane-associated protein	20	1
191813	Major facilitator superfamily	20	1
162221	Cysteine synthase A	20	1
181811	Membrane-bound transcriptional regulator LytR	20	1
31361	Transcriptional regulators containing a DNA-binding HTH domain and an aminotransferase domain (MocR family) and their eukaryotic orthologs	20	1
179521	D-serine dehydratase	20	1
188607	D-alanyl-lipoteichoic acid biosynthesis protein DltD	20	1
162128	Carboxylate/amino acid/amine transporter	20	1
110729	Collagenase	20	1
129987	Amino acid transporter	20	1
173869	The substrate-binding component of an ABC-type oligopeptide import system containing the type 2 periplasmic binding fold	19	13
163059	Germination protein, Ger(x)C family	19	7
183898	N-acetylglucosamine-binding protein A	19	6
189798	Sodium/hydrogen exchanger family	19	6
30836	Putative regulatory ligand-binding protein related to C-terminal domains of K channels	19	4
178836	L-lactate dehydrogenase	19	1
31520	Transcriptional regulators, similar to M	19	1
31856	Acetyltransferases, including N-acetylases of ribosomal proteins	19	1
48387	Nitroreductase-like family 4	19	1
176695	C-terminal domain of *Sphingobium chlorophenolicum* 2,6-dichloro-p-hydroquinone 1,2-dioxygenase (PcpA) and similar proteins	19	1
145290	BCCT family transporter	19	1
110729	Collagenase	19	1
30749	Predicted esterase	19	1
31331	ABC-type antimicrobial peptide transport system, ATPase component	19	1
147640	NosL. NosL is one of the accessory proteins of the nos (nitrous oxide reductase) gene cluster	19	1
162053	Serine transporter	19	1

### Shared genes between plasmids and chromosomes are regulated by different elements

**Figure 5:**
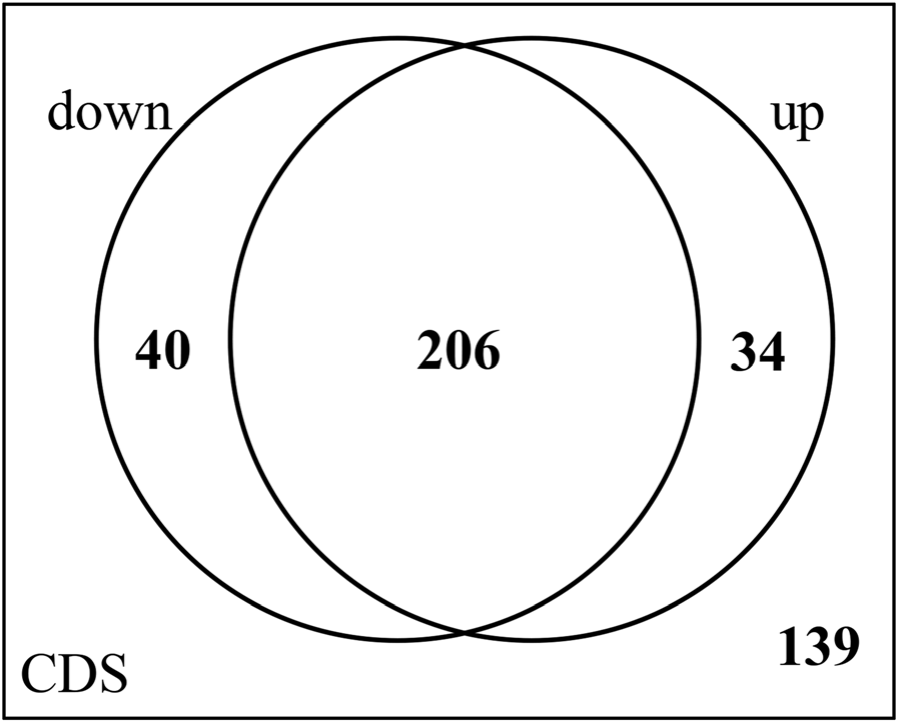
**Among the 419 gene pairs from the same host, only 139 genes had similar upstream and downstream sequences.** CDS, coding sequence of shared gene pairs; up, upstream 200-bp sequences; down, downstream 100-bp sequences.

Although many genes had been exchanged between plasmids and chromosomes, most of them had different promoters and terminators. We focused on genes shared between plasmids and the chromosome from the same host. The promoter and terminator sequences of a gene are located upstream and downstream of the coding sequence. We compared the upstream and downstream sequences of each of the 419 pairs of shared genes from the same host and found that only 139 genes had similar upstream and downstream sequences. Among the other 280 gene pairs, 240 had different upstream sequences, meaning these gene pairs had different promoters; 246 had different downstream sequences, meaning that these gene pairs had different terminators; and 206 pairs had different upstream and downstream sequences, indicating different promoters and terminators (Figure 5). This finding suggests that approximately two-thirds of the shared genes between plasmids and the chromosome from the same host are controlled at the transcriptional level by different elements.

This suggestion is supported by the reported transcriptome data for *B. thuringiensis* serovar *chinensis* CT-43 [31]. We examined this strain and focused on the 12 shared gene pairs composed of only one gene on the chromosome and one on a plasmid. We found that the shared genes with similar upstream and downstream sequences usually had similar transcriptional dynamics among the four time points, representing mid-exponential growth phase, early-stationary growth phase, mid-stationary growth phase and the time point when 30% of mother cells are lysed, whereas those with different upstream and/or downstream sequences had different transcriptional dynamics (Additional file 2: Table S4). The CT43_CH0952/CT43_P281096 gene pair, which had similar upstream and downstream sequences, had similar transcriptional dynamics during the four time points. Other shared gene pairs had different upstream and/or downstream sequences, and the genes in these pairs differed in their transcriptional dynamics.

## Discussion

Many strains of the *B. cereus* group contain several plasmids with different replicons, and these plasmids have different genome sizes, ranging from 2 to 600 kb. However, the functions determined by these plasmids have rarely been considered; only those with apparent pathogenic features have been well studied, such as the function of the two plasmids of *B. anthracis* that contribute to anthrax disease [21], the function of the emetic *B. cereus* plasmids that determine the emetic syndrome and the functions of some *B. thuringiensis* plasmids that cause toxicity to insects [21,24]. The other plasmids have not been well studied, although they represent the majority of the *B. cereus* group plasmids. This study focused on the pan-genome of the entire group.

We considered all of the plasmids as a group. Genes contained by plasmids were found to be similar to chromosomal variable genes but different from chromosomal core genes in terms of the basic features and the functions they provide. Genes on plasmids and chromosomal variable genes were considered non-essential genes, and they only differed in their location. However, genes on plasmids showed some differences from chromosomal variable genes; the former were enriched in functions of transcriptional regulation, but the latter were not (Additional file 3: Figure S1). This finding indicates that plasmids show some differences from chromosomes in the functions of the genes they harbor. However, all of the functional categories determined by the chromosomal core genes were presented by genes on plasmids. This finding indicates that genes with functions in basic metabolism and even some genes with essential functions for bacterial survival were also present on plasmids. This phenomenon has also been reported in other bacteria: nearly 11% of the genes on plasmid p42e of *Rhizobium etli* CFN42 participate in primary metabolism [32]. We predict that for the entire group, many of the genes that function in basic metabolism are present as two copies, one on the chromosome and the other on a plasmid. However, some essential genes were never found on plasmids within this group, such as genes for different types of ribosomal RNA; this differentiated the plasmids from the chromosomes.

Genetic exchange events have occurred frequently between plasmids and chromosomes, and almost all the regions of the chromosome were affected by these events. Moreover, because some of these events occurred along with the formation of the species, we predict that some plasmids of this group have long histories and were obtained by their hosts prior to lineage formation. During the long course of evolutionary history, many genes were harbored on both plasmids and chromosomes. For the entire group or even for certain strains, this led to the duplication of some genes and caused redundancies in many functions. These redundancies are only present at the DNA level because homologous genes between chromosomes and plasmids usually have different promoters and terminators, indicating that they are controlled by different regulatory elements at the transcriptional level. Moreover, gene families that function as transcriptional regulators showed much greater abundance on plasmids, and they could be involved in the control of genes on plasmids. Data from transcriptomics and proteomics studies have indicated that genes on chromosomes are more active than those on plasmids [31,33]. Moreover, shared genes in the same cell have higher levels of transcription and translation on plasmids than on chromosomes. We suggest that genes on plasmids are more strictly controlled by regulators, which could neutralize the redundancies caused by homologous genes.

To survive in varied environments (soil, water, and animal hosts), members of the *B. cereus* group employ HGT to take up different types of genes that assist in adaptation and can integrate these genes into chromosomes or plasmids [21,34-36]. When a strain has existed in a steady environment for a long time, some essential genes may be integrated into the chromosome, whereas non-essential genes must be controlled more strictly or even lost. However, for the entire group, as the environment changes frequently, adaptive genes must be preserved on plasmids or chromosomes. This practice contributes to the survival of members of this group in different types of environments.

This study focused on the shared genes between plasmids and chromosomes, which provides somewhat incomplete evidence for the above conclusion. More analyses based on genome information and more laboratory experiments testing these deductions are needed in future work.

## Conclusion

All of the plasmids were transferred frequently among members of the group and mediated numerous genetic exchange events among plasmids and between plasmids and chromosomes. For the entire group, most genes were located on both plasmids and chromosomes, with the copies on plasmids being more strictly controlled. We suggest that plasmids are vectors for redundant genes on chromosomes.

## Methods

### Sequence collection

The genome sequences of 104 plasmids (80 from GenBank and 24 from our group) were used in the analyses. The genome sizes of these 104 plasmids ranged from ~2 kb to ~566 kb (Additional file 4: Table S1). The sequences of 20 chromosomes (18 from GenBank and 2 from our group) were used for the shared gene analysis (Additional file 4: Table S2).

### Gene clustering

Protein sequences longer than 50 amino acids from all chromosomes and plasmids were searched using BLASTP [37] with an all-against-all style and the default parameters. Protein sequences with identities and coverage greater than 70% were then clustered into families using the program OrthoMCL with an inflation value of 2 [38].

All the start positions and end positions of shared genes on a plasmid or a chromosome were compared against a series of uniform randomly distributed numbers with the same length as the positions to determine whether the positions showed a uniform random distribution on the plasmid and chromosome. All the analyses were conducted in R [39].

### COGs, base composition, codon usage and operon analysis

To identify chromosomal core genes and chromosomal distributed genes, one gene per family was randomly extracted from chromosomal clusters derived from *B. cereus* strains whose complete genome sequences were available. Unique genes from each chromosome of the above strains were combined to form chromosomal unique genes. Moreover, the plasmid distributed genes consisted of one random gene per family together with all the unique genes from all the plasmids whose genome sequences were available.

For the COG analysis, we constructed a local COG database [40] and ran RPSBLAST [37] using the sequence sets described above as queries with an e-value cutoff of 0.001. We focused on the top three hits from each alignment and counted each category for comparison using an in-house Perl script. The base composition was analyzed using G-language [41], and a CAI (codon adaptation index) analysis was performed using codonW software (version 1.4.4, http://codonw.sourceforge.net/).

The operons were predicted by ProOpDB [42]. To compare the promoters and terminators of shared genes between chromosomes and plasmids, we collected 200-bp upstream and 100-bp downstream sequences for each coding region of all these shared gene pairs. Then, we compared these sequences using BLAST.

### Phylogenetic tree construction

Each of the 40 families of sequences of genes shared between plasmids and the chromosomal core set were used for phylogenetic tree construction. A maximum likelihood tree was generated by the PhyML software [43] with bootstrap support calculated from 100 replicates after each group of sequences was aligned by Muscle [44].

All the statistical analyses were performed using in-house Perl scripts and R 2.15.1 [39].

### Availability of supporting data

The data sets supporting the results of this article are included within the article and the additional files.

## Additional files

Additional file 1: Table S3. The 598 gene families shared by plasmids and chromosomes. Additional file 2: Table S4. Transcriptional profiles of shared genes between plasmids and chromosome that have similar promoter and terminator sequences among the four time points. Additional file 3: Figure S1. The proportions of each COG category for the chromosomal core gene set, chromosomal distributed gene set, chromosomal unique gene set, plasmids and chromosomal shared gene set as well as all the genes on plasmids. Additional file 4:
**Table S1.** Plasmids analyzed in this study. **Table S2** Genomes used in this study.
