# Supplementary figures and images for: Plasmids are vectors for redundant chromosomal genes in the Bacillus cereus group
Source: BMC Genomics. 2015 Jan 22;16(1):6. doi: 10.1186/s12864-014-1206-5 (PMC4326196; doi:10.1186/s12864-014-1206-5)

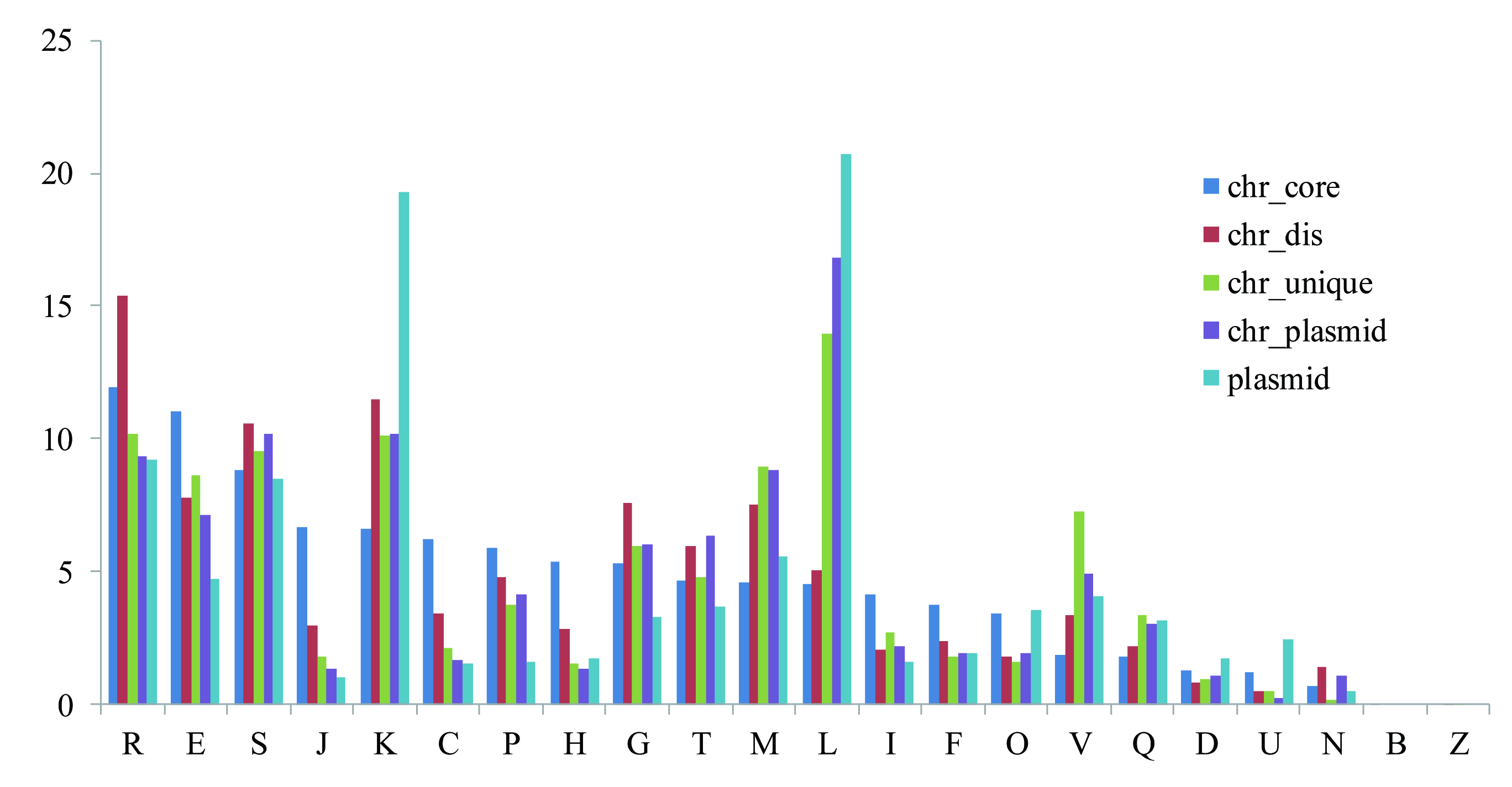

Supplement: Additional file 3: Figure S1. — The proportions of each COG category for the chromosomal core gene set, chromosomal distributed gene set, chromosomal unique gene set, plasmids and chromosomal shared gene set as well as all the genes on plasmids. [file 12864_2014_1206_MOESM3_ESM.tiff]
