# Supplementary material for: Plasmids are vectors for redundant chromosomal genes in the Bacillus cereus group
Source: BMC Genomics. 2015 Jan 22;16(1):6. doi: 10.1186/s12864-014-1206-5 (PMC4326196; doi:10.1186/s12864-014-1206-5)
Supplement: Additional file 4: — Table S1. Plasmids analyzed in this study. Table S2 Genomes used in this study. [file 12864_2014_1206_MOESM4_ESM.doc]

**Table S1** Plasmids analyzed in this study

| plasmid | Host strain | GC% | Gene | Size (bp) | Accession number | Reference |
| --- | --- | --- | --- | --- | --- | --- |
| pXO1 | *Ba* Ames Ancestor | 32.5 | 201 | 181,677 | NC_007322 |  |
| pXO2 |  | 33 | 109 | 94,830 | NC_007323 |  |
| p03BB102_179 | *Bc* 03BB102 | 32.2 | 209 | 179,680 | NC_012473 |  |
| p03BB108_10 | *Bc* 03BB108 | 31.8 | 18 | 9,797 | NZ_ABDM02000066 |  |
| p03BB108_42 |  | 36.1 | 70 | 42,470 | NZ_ABDM02000065 |  |
| p03BB108_86 |  | 32.5 | 106 | 85,879 | NZ_ABDM02000064 |  |
| p03BB108_239 |  | 31.9 | 227 | 238,933 | NZ_ABDM02000063 |  |
| p03BB108_282 |  | 30.8 | 333 | 282,009 | NZ_ABDM02000062 |  |
| pBClin15 | *Bc* 14579 | 38 | 21 | 15,274 | NC_004721 |  |
| pAH1134_14 | *Bc* AH1134 | 30.6 | 17 | 14,812 | NZ_ABDA02000037 |  |
| pAH1134_566 |  | 32.3 | 712 | 565,964 | NZ_ABDA02000035  NZ_ABDA02000036 |  |
| pAH187_3 | *Bc* AH187 | 34.9 | 5 | 3,091 | NC_011657 |  |
| pAH187_12 |  | 31.1 | 23 | 12,481 | NC_011654 |  |
| pAH187_45 |  | 35.5 | 60 | 45,173 | NC_011656 |  |
| pAH187_270 |  | 34.2 | 277 | 270,082 | NC_011655 |  |
| pPER272 | *Bc* AH818 | 33.6 | 271 | 272,145 | DQ889678 |  |
| pAH820_3 | *Bc* AH820 | 34.9 | 5 | 3,091 | NC_011776 |  |
| pAH820_10 |  | 33.6 | 11 | 10,915 | NC_011771 |  |
| pAH820_272 |  | 33.6 | 321 | 272,145 | NC_011777 |  |
| pBc10987 | *Bc* ATCC 10987 | 33.4 | 242 | 208,369 | NC_005707 |  |
| pBC210 | *Bc* G9241 | 31.7 | 201 | 209,385 | NC_010933 |  |
| pBCXO1 |  | 32.6 | 186 | 190,861 | NC_010934 |  |
| pCI-XO1 | *Bc* CI | 32.5 | 214 | 181,907 | NC_014331 |  |
| pCI-XO2 |  | 33.1 | 110 | 94,469 | NC_014332 |  |
| pBAslCI14 |  | 37.9 | 22 | 14,219 | NC_014333 |  |
| pE33L5 | *Bc* E33L | 30.9 | 5 | 5,108 | NC_007104 |  |
| pE33L8 |  | 31.9 | 8 | 8,191 | NC_007106 |  |
| pE33L9 |  | 31 | 10 | 9,150 | NC_007107 |  |
| pE33L54 |  | 31.9 | 57 | 53,501 | NC_007105 |  |
| pE33L466 |  | 33.1 | 447 | 466,370 | NC_007103 |  |
| pG9842_140 | *Bc* G9842 | 32.9 | 120 | 140,001 | NC_011774 |  |
| pG9842_209 |  | 30 | 251 | 209,488 | NC_011775 |  |
| pBc239 | *Bc* Q1 | 33.5 | 228 | 239,246 | NC_011973 |  |
| pBc53 |  | 35.1 | 69 | 52,766 | NC_011971 |  |
| pH308197_3 | *Bc* H3081.97 | 34.6 | 5 | 3,424 | NC_011338 |  |
| pH308197_10 |  | 30.1 | 12 | 10,077 | NC_011341 |  |
| pH308197_11 |  | 32.9 | 16 | 11,567 | NC_011340 |  |
| pH308197_29 |  | 32.3 | 40 | 29,189 | NC_011342 |  |
| pH308197_73 |  | 30.2 | 101 | 72,792 | NC_011337 |  |
| pH308197_258 |  | 34.1 | 257 | 258,484 | NC_011339 |  |

**Table S1** continued.

| plasmid | Host strain | GC% | Gene | Size (bp) | Accession number | Reference |
| --- | --- | --- | --- | --- | --- | --- |
| pPRS3a | Bc PRS3 | 54.3 | 2 | 4,126 | GQ404376 |  |
| pLVP1401 | *Bc* VPC1401 | 31.1 | 65 | 56,149 | NC_014757 |  |
| pBC9801 | *Bcy* NVH 391-98 | 30.3 | 11 | 7,135 | NC_009673 |  |
| pBMYdx | *Bm* DXv | 35.4 | 2 | 3,377 | AJ272266 |  |
| pDx14.2 |  | 29.5 | 14 | 14,218 | NC_006870 |  |
| pBMY1 | *Bm* SINv | 35.4 | 1 | 3,476 | AJ243967 |  |
| pSin9.7 |  | 32.4 | 7 | 9,698 | NC_006869 |  |
| pBtoxis | *Bt* 4Q5 | 32.4 | 124 | 127,923 | NC_010076 |  |
| pBT9727 | *Bt* 97-27 | 32.6 | 80 | 77,112 | NC_006578 |  |
| pALH1 | *Bt* Al Hakam | 36.2 | 62 | 55,939 | NC_008598 |  |
| pBMB171 | *Bt* BMB171 | 33.3 | 276 | 312,963 | NC_014172 |  |
| pCT6880 | *Bt* CT-43 | 31.8 | 6 | 6,880 | CP001912 |  |
| pCT8252 |  | 32.4 | 8 | 8,252 | CP001914 |  |
| pCT8513 |  | 30.8 | 10 | 8,513 | CP001916 |  |
| pCT9547 |  | 33.1 | 5 | 9,547 | CP001917 |  |
| pCT14 |  | 31.5 | 22 | 14,860 | CP001909 |  |
| pCT51 |  | 35 | 72 | 51,488 | CP001911 |  |
| pCT72 |  | 32 | 86 | 72,074 | CP001913 |  |
| pCT83 |  | 33.2 | 98 | 83,590 | CP001915 |  |
| pCT127 |  | 32.1 | 141 | 127,885 | CP001908 |  |
| pCT281 |  | 33 | 279 | 281,231 | CP001910 |  |
| pBMB2062 | *Bt* HD1 | 34.8 | 3 | 2,062 | - |  |
| pBMB7635 |  | 32.2 | 10 | 7,635 | - |  |
| pBMB8240 |  | 29.7 | 11 | 8,240 | - |  |
| pBMB8513 |  | 30.9 | 10 | 8,513 | - |  |
| pBMB14721 |  | 31 | 23 | 14,721 | - |  |
| pBMB14870 |  | 40.1 | 25 | 14,870 | - |  |
| pBMB46 |  | 35.4 | 67 | 46,634 | - |  |
| pBMB64 |  | 31.9 | 75 | 64,522 | - |  |
| pBMB65 |  | 34.8 | 62 | 65,873 | - |  |
| pBMB74 |  | 33.7 | 82 | 74,480 | - |  |
| pBMB95 |  | 31.5 | 102 | 95,983 | - |  |
| pBMB302 |  | 33.3 | 314 | 302,255 | - |  |
| pBMB431 |  | 32.7 | 380 | 431,971 | - |  |
| pAW63 | *Bt* HD73 | 33.8 | 75 | 71,777 | NC_010599 |  |
| pFR12 | *Bt* INTA-FR7-4 | 31.2 | 11 | 12,095 | NC_010281 |  |
| pFR12.5 |  | 29.4 | 14 | 12,459 | EU362918 |  |
| pFR55 |  | 33.7 | 69 | 55,712 | NC_010283 |  |
| pBMBt1 | *Bt* INTA Mo14-4 | 32.4 | 3 | 6,700 | AY822042 |  |
| pK1S1 | *Bt* K1 | 32.8 | 7 | 5,475 | NC_009034 |  |
| pUIBI-1 | *Bt* LBIT-113 | 32.2 | 4 | 4,671 | AF516904 |  |
| pTX14-1 | *Bt* subsp. *israelensis* | 36.3 | 3 | 5,415 | NC_002091 |  |

**Table S1** continued.

| plasmid | Host strain | GC% | Gene | Size (bp) | Accession number | Reference |
| --- | --- | --- | --- | --- | --- | --- |
| pTX14-2 | *Bt* subsp. *israelensis* | 36 | 3 | 6,829 | AY138808 |  |
| pTX14-3 | *Bt* subsp. *israelensis* | 35.2 | 2 | 7,649 | NC_001446 |  |
| pGI1 | *Bt* subsp. *thuringiensis* | 32.4 | 4 | 8,254 | AY138809 |  |
| pGI3 | *Bt* subsp. *thuringiensis* | 31.7 | 11 | 11,365 | NC_005567 |  |
| pBMB26 | *Bt* YBT-020 | 33.1 | 192 | 187,880 | CP002509 |  |
| pBMB28 |  | 33.9 | 147 | 139,013 | CP002510 |  |
| pBMB2062 | *Bt* YBT-1520 | 34.8 | 2 | 2,062 | AF050161 |  |
| pBMB7635 |  | 32.2 | 11 | 7,635 | NC_011796 |  |
| pBMB7921 |  | 32.3 | 12 | 7,921 | - |  |
| pBMB8240 |  | 29.7 | 13 | 8,240 | - |  |
| pBMB8513 |  | 30.8 | 12 | 8,513 | - |  |
| pBMB9741 |  | 32.4 | 2 | 6,578 | AF202532 |  |
| pBMB11769 |  | 31.8 | 19 | 11,769 | - |  |
| pBMB67 |  | 32.4 | 73 | 67,159 | NC_009841 |  |
| pBMB137 |  | 32.5 | 156 | 137,573 | - |  |
| pBMB293 |  | 33.2 | 299 | 293,574 | - |  |
| pBMB400 |  | 32.5 | 385 | 416,210 | - |  |
| pBMB175 | *Bt* YBT-1765 | 31 | 13 | 14,841 | NC_010895 |  |
| pBWB401 | *Bw* KBAB4 | 33.7 | 332 | 417,054 | NC_010180 |  |
| pBWB402 |  | 33.3 | 75 | 75,107 | NC_010181 |  |
| pBWB403 |  | 43.4 | 76 | 64,977 | NC_010182 |  |
| pBWB404 |  | 35.4 | 71 | 52,830 | NC_010183 |  |

-: unpublished data, *Ba*: *Bacilluse anthracis*, *Bc*: *Bacillus cereus*, *Bcy*: *Bacillus cytotoxicus*, *Bm*: *Bacillus mycoides*, *Bt*: *Bacillus thuringiensis*, *Bw*: *Bacillus weihenstephanensis*

**Table S2** Genomes used in this study

| Strain name | Species | Plasmids | Size (Mb) | GC | Acc. number | Reference |
| --- | --- | --- | --- | --- | --- | --- |
| Ames Ancestor | *Ba* | 2 | 5.23 | 35.3 | NC_007530 |  |
| 03BB102 | *Bc* | 1 | 5.27 | 35.3 | NC_012472 |  |
| AH187 | *Bc* | 4 | 5.27 | 35.5 | NC_011658 |  |
| AH820 | *Bc* | 3 | 5.3 | 35.3 | NC_011773 |  |
| ATCC 10987 | *Bc* | 1 | 5.22 | 35.5 | NC_003909 |  |
| ATCC 14579 | *Bc* | 1 | 5.42 | 35.3 | NC_004722 |  |
| B4264 | *Bc* | 0 | 5.42 | 35.2 | NC_011725 |  |
| biovar anthracis str. CI | *Bc* | 3 | 5.2 | 35.3 | NC_014335 |  |
| E33L | *Bc* | 5 | 5.3 | 35.2 | NC_006274 |  |
| G9842 | *Bc* | 2 | 5.39 | 35 | NC_011772 |  |
| Q1 | *Bc* | 2 | 5.21 | 35.5 | NC_011969 |  |
| NHV 391-98 | *Bcy* | 1 | 4.09 | 35.9 | NC_009674 |  |
| Al Hakam | *Bt* | 1 | 5.26 | 35.4 | NC_008600 |  |
| BMB171 | *Bt* | 5 | 5.33 | 35.2 | NC_014171 |  |
| subsp. *chinensis* CT-43 | *Bt* | 10 | 5.49 | 35.1 | NC_017208 |  |
| subsp. *finitimus* YBT-020 | *Bt* | 2 | 5.36 | 35.4 | NC_017200 |  |
| subsp. *konkukian* str. 97-27 | *Bt* | 1 | 5.24 | 35.4 | NC_005957 |  |
| HD1 | *Bt* | 13 | 5.63 | 35.3 | - |  |
| YBT-1520 | *Bt* | 11 | 5.6 | 35.3 | - |  |
| KBAB4 | *Bw* | 4 | 5.26 | 35.6 | NC_010184 |  |

-: unpublished data, *Ba*: *Bacillus anthracis*, *Bc*: *Bacillus cereus*, *Bcy*: *Bacillus cytotoxicus*, *Bt*: *Bacillus thuringiensis*, *Bw*: *Bacillus weihenstephanensis*

**Supplementary References**

Amadio AF, Benintende GB, Zandomeni RO. 2009. Complete sequence of three plasmids from *Bacillus thuringiensis* INTA-FR7-4 environmental isolate and comparison with related plasmids from the *Bacillus cereus* group. *Plasmid* 62: 172-182.

Andrup L, Jensen GB, Wilcks A, Smidt L, Hoflack L, Mahillon J. 2003. The patchwork nature of rolling-circle plasmids: comparison of six plasmids from two distinct *Bacillus thuringiensis* serotypes. *Plasmid* 49: 205-232.

Berry C, O'Neil S, Ben-Dov E, Jones AF, Murphy L, Quail MA, Holden MT, Harris D, Zaritsky A, Parkhill J. 2002. Complete sequence and organization of pBtoxis, the toxin-coding plasmid of *Bacillus thuringiensis* subsp. *israelensis*. *Appl Environ Microbiol.* 68: 5082-5095.

Challacombe JF, Altherr MR, Xie G, et al. . 2007. The complete genome sequence of *Bacillus thuringiensis* Al Hakam. *J Bacteriol.* 189: 3680-3681.

Chao L, Qiyu B, Fuping S, Ming S, Dafang H, Guiming L, Ziniu Y. 2007. Complete nucleotide sequence of pBMB67, a 67-kb plasmid from *Bacillus thuringiensis* strain YBT-1520. *Plasmid* 57: 44-54.

Di Franco C, Pisaneschi G, Beccari E. 2000. Molecular analysis of two rolling-circle replicating cryptic plasmids, pBMYdx and pBMY1, from the soil gram-positive *Bacillus mycoides*. *Plasmid* 44: 280-284.

Di Franco C, Santini T, Pisaneschi G, Beccari E. 2005. Insights into the genetic organization of the *Bacillus mycoides* cryptic plasmids pDx14.2 and pSin9.7 deduced from their complete nucleotide sequence. *Plasmid* 54: 288-293.

Han CS, Xie G, Challacombe JF, et al. (48 co-authors). 2006. Pathogenomic sequence analysis of *Bacillus cereus* and *Bacillus thuringiensis* isolates closely related to *Bacillus anthracis*. *J Bacteriol.* 188: 3382-3390.

He J, Shao X, Zheng H, et al. (11 co-autors). 2010. Complete genome sequence of *Bacillus thuringiensis* mutant strain BMB171. *J Bacteriol* 192: 4074-4075.

He J, Wang J, Yin W, Shao X, Zheng H, Li M, Zhao Y, Sun M, Wang S, Yu Z. 2011. Complete genome sequence of *Bacillus thuringiensis* subsp. *chinensis* strain CT-43. *J Bacteriol.* 193: 3407-3408.

Huang J, Han D, Yu Z, Sun M. 2007. A novel cryptic plasmid pBMB175 from *Bacillus thuringiensis* subsp. *tenebrionis* YBT-1765. *Arch Microbiol* 188: 47-53.

Ivanova N, Sorokin A, Anderson I, et al. . 2003. Genome sequence of *Bacillus cereus* and comparative analysis with *Bacillus anthracis*. *Nature* 423: 87-91.

Klee SR, Brzuszkiewicz EB, Nattermann H, et al. (18 co-authors). 2010. The genome of a *Bacillus* isolate causing anthrax in chimpanzees combines chromosomal properties of *B. cereus* with *B. anthracis* virulence plasmids. *PLoS One* 5: e10986.

Lapidus A, Goltsman E, Auger S, et al. (18 co-authors). 2008. Extending the *Bacillus cereus* group genomics to putative food-borne pathogens of different toxicity. *Chem Biol Interact.* 171: 236-249.

Liu X, Zhu S, Ye W, Ruan L, Yu Z, Zhao C, Sun M. 2008. Genetic characterization of two putative toxin-antitoxin systems on cryptic plasmids from *Bacillus thuringiensis* strain YBT-1520. *J Microbiol Biotechnol.* 18: 1630-1633.

Loeza-Lara PD, Benintende G, Cozzi J, Ochoa-Zarzosa A, Baizabal-Aguirre VM, Valdez-Alarcon JJ, Lopez-Meza JE. 2005. The plasmid pBMBt1 from *Bacillus thuringiensis* subsp. *darmstadiensis* (INTA Mo14-4) replicates by the rolling-circle mechanism and encodes a novel insecticidal crystal protein-like gene. *Plasmid* 54: 229-240.

Lopez-Meza JE, Barboza-Corona JE, Del Rincon-Castro MC, Ibarra JE. 2003. Sequencing and characterization of plasmid pUIBI-1 from *Bacillus thuringiensis* serovar *entomocidus* LBIT-113. *Curr Microbiol.* 47: 395-399.

Rasko DA, Ravel J, Okstad OA, et al. (15 co-authors). 2004. The genome sequence of *Bacillus cereus* ATCC 10987 reveals metabolic adaptations and a large plasmid related to *Bacillus anthracis* pXO1. *Nucleic Acids Res.* 32: 977-988.

Rasko DA, Rosovitz MJ, Okstad OA, Fouts DE, Jiang L, Cer RZ, Kolsto AB, Gill SR, Ravel J. 2007. Complete sequence analysis of novel plasmids from emetic and periodontal *Bacillus cereus* isolates reveals a common evolutionary history among the *B. cereus*-group plasmids, including *Bacillus anthracis* pXO1. *J Bacteriol.* 189: 52-64.

Ravel J, Jiang L, Stanley ST, et al. (11 co-authors). 2009. The complete genome sequence of *Bacillus anthracis* Ames "Ancestor". *J Bacteriol.* 191: 445-446.

Van der Auwera GA, Andrup L, Mahillon J. 2005. Conjugative plasmid pAW63 brings new insights into the genesis of the Bacillus anthracis virulence plasmid pXO2 and of the *Bacillus thuringiensis* plasmid pBT9727. *BMC Genomics* 6: 103.

Xiong Z, Jiang Y, Qi D, et al. (12 co-authors). 2009. Complete genome sequence of the extremophilic *Bacillus cereus* strain Q1 with industrial applications. *J Bacteriol.* 191: 1120-1121.

Zhang Q, Sun M, Xu Z, Yu Z. 2007. Cloning and characterization of pBMB9741, a native plasmid of *Bacillus thuringiensis* subsp. *kurstaki* strain YBT-1520. *Curr Microbiol.* 55: 302-307.

Zhu Y, Shang H, Zhu Q, et al. (14 co-authors). 2011. Complete genome sequence of *Bacillus thuringiensis* serovar *finitimus* strain YBT-020. *J Bacteriol.* 193: 2379-2380.
